# Dynamics of the pneumonic plague epidemic in Madagascar, August to October 2017

**DOI:** 10.2807/1560-7917.ES.2017.22.46.17-00710

**Published:** 2017-11-16

**Authors:** Shinya Tsuzuki, Hyojung Lee, Fuminari Miura, Yat Hin Chan, Sung-mok Jung, Andrei R Akhmetzhanov, Hiroshi Nishiura

**Affiliations:** 1 Graduate School of Medicine, Hokkaido University, Sapporo, Japan; 2 Graduate School of Engineering, The University of Tokyo, Tokyo, Japan

**Keywords:** Outbreak, *Yersinia pestis*, Basic reproduction number, Case fatality ratio, importation, risk assessment

## Abstract

Transmission potential and severity of pneumonic plague in Madagascar were assessed. Accounting for reporting delay, the reproduction number was estimated at 1.73. The case fatality risk was estimated as 5.5%. Expected numbers of exported cases from Madagascar were estimated across the world and all estimates were below 1 person from August to October, 2017.

While plague in Madagascar has been recognised as endemic for more than two decades [1-3], the country has experienced the largest ever observed epidemic of pneumonic plague in 2017 through human-to-human transmission [4]. As of 31 October 2017, the cumulative total numbers of 1,838 cases and 64 deaths have been reported [5]. To guide risk assessment, it is vital to quantitatively characterise the risks of secondary transmission, fatal outcome given infection and exporting the disease from Madagascar to elsewhere. We statistically estimate these risks by analysing the epidemiological data in real time.

## Epidemiological data

**Figure 1:**
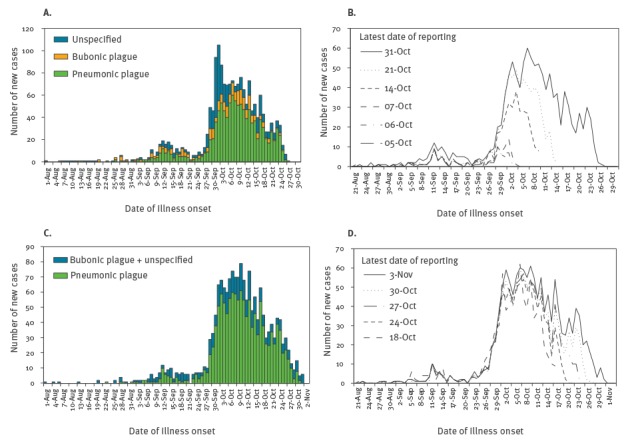
Epidemic curves of plague cases in Madagascar, August – October 2017

Three datasets were obtained. First, the epidemiological bulletin of the Institut Pasteur de Madagascar (IPM) was explored to retrieve the epidemic curve of the sum of suspected, probable and confirmed cases that are stratified by clinical form of plague (i.e. bubonic plague, pneumonic plague and unspecified). Second, in addition to the dataset from IPM, we also analysed the data reported by the World Health Organization Regional Office for Africa (AFRO) [5,6]. The definition of clinical form adhered to the original distinction, i.e. bubonic plague is characterised by fever, painful bubo (lymphadenopathy) at inguinal, femoral, axillary, cervical, or submaxillary nodes, while pneumonic plague is characterised by fever, cough, chest pain and bloody sputum [5]. For each of the IPM and AFRO datasets, we used different epidemic curves of pneumonic plague (Figure 1). The first was the epidemic curve for which we assumed that a constant growth rate (or a constant reproduction number) was maintained. For the IPM dataset, this was in a report from 21 October 2017, while for the AFRO dataset, this was in a report from 24 October 2017. Both reports had data up to 17 October 2017. The latest epidemic curves of IPM and AFRO as of 31 October 2017 and 3 November, respectively, are shown in Figures 1A and 1C, but our estimations were conducted on 24 October 2017 and used one earlier report for each data source. In these reports, the number of cases in recent days was likely underestimated due to reporting delay. The other epidemic curves considered in our study (Figures 1B and 1D) were multiple epidemic curves of pneumonic plague, each compiled at a successive time point during the outbreak for reporting purposes. These curves overlap to a certain extent, but in general a higher number of cases at a given calendar date may be observed in the curves compiled/updated later in the outbreak. This is a reflection of the reporting delay, which is retrospectively addressed with time as more data become available. In addition to counts of cases, we also obtained reported numbers of deaths at multiple reporting dates from IPM. That is, on 3, 4, 5, 12, 15, 17 and 31 October 2017, there have been 9, 9, 9, 22, 23, 27 and 48 deaths among the total of 67, 86, 106, 353, 512, 573 and 1,138 cases, respectively.

(A) and (C) The epidemic curves of plague by clinical form obtained from the Institut Pasteur de Madagascar (IPM) [5] and the World Health Organization Regional Office for Africa (AFRO) [6], respectively. These are the latest available epidemic curves of the plague epidemic in Madagascar as of 31 October 2017 for IPM and 3 November 2017 for AFRO (the latest access to the most updated data). Using earlier epidemic curves (reported on 21 October and 24 October 2017 for IPM and AFRO, respectively) and implementing statistical estimation on 24 October 2017, *R*_0_ was estimated, assuming that there was a constant growth rate (i.e. a constant reproduction number) by 17 October 2017. (B) (D) multiple epidemic curves of pneumonic plague cases reported by IPM and AFRO respectively at different time points of the outbreak. Line graphs show the latest number of cases at the date of reporting. Reports are updated as time goes on, and the count of cases on the same day is also modified. The vertical differences between lines on the same illness onset date reflects the delay in reporting.

The third dataset consisted of population data for Madagascar that was obtained from the World Bank [7], as well as inbound and outbound travel volumes retrieved from the Ministry of Tourism in Madagascar [8] and the World Tourism Organization (UNWTO) [9], the latter including business travellers in the counts of travellers. The average duration of stay of inbound international travellers in Madagascar was obtained from the World Bank [7].

## Epidemiological modelling

Here we estimate three key quantities using mathematical models, i.e. (i) the basic reproduction number, *R*_0_, the average number of secondary cases generated by a single primary case, (ii) the case fatality risk (CFR), i.e. the conditional risk of death given diagnosis as case, and (iii) the expected number of pneumonic plague cases departing from Madagascar to each country, comprising importations by visitors to Madagascar as they return to their country of residence and exportations by local resident of Madagascar as they travel abroad. Assuming that the current pneumonic plague epidemic has been driven by an index case [4,10], we focus on pneumonic plague data and assume that all cases of pneumonic plague were generated by human-to-human transmission (i.e. primary pneumonic plague).

### Basic reproduction number

To estimate *R*_0_, we employed the renewal equation. Let *j*_t_ be the number of new cases on day *t*, we have

$E(j_{t})=R_{0}\sum_{\tau =1}^{t-1}j_{t-\tau }g_{\tau }$, (1)

where *g*_τ_ represents the discretised distribution of the serial interval derived as *g*_τ_ = *G*(*τ*) − *G*(*τ* − 1) for *τ* > 0 with mean and variance at 5.1 days and 2.3 day^2^ for *G*(*τ*) (i.e. a gamma distribution with shape and scale parameters at 5.4 and 0.9, respectively) [11]. Day 0 was set to be 23 August 2017 on which the index case developed fever (and here we discard infectiousness before the illness onset). Assuming that the observed incidence followed a Poisson distribution with the expected value in the right-hand side of (1), we estimated *R*_0_ using the single epidemic curve (obtained from IPM on 21 October and from AFRO on 24 October, respectively) assuming that the constant growth rate was applicable by 17 October 2017. The estimation was conducted by independently using IPM and AFRO data. However, since the latest cases in that curve were likely under-reported due to reporting delay, we truncated the epidemic curve by removing the information of the latest cases from our analysis for 0–7 recent days (assuming that cases in recent 0–7 days were under-reported due to reporting delay).

We also estimated *R*_0_ from multiple curves (Figure 1B) provided by IPM, while jointly quantifying the reporting delay distribution. In this analysis, we did not explore the dataset from AFRO because it contained some discrepancies of values among the curves (i.e. the number of cases declined as a function of reporting date). Let *c*_t,x_ be daily reported incidence on day *t* with the reporting date on day *x*. Then, the reported incidence is expressed by using the renewal equation as follows. Let *F*(*t*) be the cumulative distribution function of the time from illness onset to reporting, which is assumed to follow a gamma distribution. The observed cases on day *t* are modelled as

$c_{t,x}=j_{t}F(x-t)$. (2)

Thus, we have

$E(c_{t,x})=\frac{c_{t,x'}}{F(x'-t)}F(x-t)$, (3)

where *x*’ is the reporting date of another epidemic curve. Let us rewrite the expected value of cases on day *t* with reporting date on day *x* as $\Lambda_{t,x}(R_{0},\;\theta )=E(c_{t,x})$. To estimate parameters governing *F*(*x*-*t*), we assume that *c_t,x_* follows a Poisson distribution:

$L(\theta ;c_{t,x})=\prod_{j=1}^{m}\prod_{t=1}^{x(j)-1}\left(\frac{e^{-\Lambda_{t,x(j)}(\theta )}\Lambda_{t,x(j)}{\left(\theta \right)}^{c_{t,x(j)}}}{c_{t,x(j)}!}\right)$, (4)

where *θ* stands for the population parameter of *F*(*x*-*t*), *x*(*j*) is the latest calendar date of reporting for an epidemic curve *j*, and *m* is the number of available epidemic curves (*m* = 6). Subsequently, we model the data generating process of the observed epidemic curve and renewal process using the following renewal equation:

$E[c_{t,x_{\max}}]=R_{0}\sum_{\tau =1}^{t-1}c_{t-\tau ,x_{\max}}g_{\tau }\frac{F(x_{\max}-t)}{F(x_{\max}-t+\tau )}$, (5)

where *x*_max_ was 17 October 2017 (i.e. the latest day by which the constant reproduction number was assumed). Let$\Lambda_{t,x_{\max}}(R_{0})=E(c_{t,x_{\max}})$. The likelihood function to estimate *R*_0_ was

$L(R_{0};c_{t,x_{\max}})=\prod_{t=1}^{x_{\max}}\left(\frac{e^{-\Lambda_{t,x_{\max}}(R_{0})}\Lambda_{t,x_{\max}}{\left(R_{0}\right)}^{c_{t,x_{\max}}}}{c_{t,x_{\max}}!}\right)$. (6)

The maximum likelihood estimate of *R*_0_ was obtained by minimising the negative logarithm of (6).

### Case fatality risk

To estimate the CFR, we account for the delay from illness onset to death, *h*_s_, which was assumed as given by *h*_s_ = *H*(*s*) − *H*(*s* − 1) for *s* > 0 where *H*(*s*) follows an exponential distribution with mean 2.3 days [12]. For seven different time points with observation (*t*_i_, where *i* = 1, 2, .., 7), the cumulative number of deaths, *D*_ti_ was reported. Let *π* be the parameter representing the unbiased CFR, the likelihood function to estimate *π* is

$L(\pi ;j_{t},\theta )=\prod_{t_{i}}\left(\begin{array}{l} \sum_{t=1}^{t_{i}}j_{t}\\ D_{t_{i}} \end{array}\right){\left(\pi \frac{\sum_{t=2}^{t_{i}}\sum_{s=1}^{t-1}j_{t-s}h_{s}}{\sum_{t=1}^{t_{i}}j_{t}}\right)}^{D_{t_{i}}}{\left(1-\pi \frac{\sum_{t=2}^{t_{i}}\sum_{s=1}^{t-1}j_{t-s}h_{s}}{\sum_{t=1}^{t_{i}}j_{t}}\right)}^{\sum_{t=1}^{t_{i}}j_{t}-D_{t_{i}}}$. (7)

The maximum likelihood estimate of the CFR was obtained by minimising the negative logarithm of (7).

### Expected number of cases due to travel to and from Madagascar

Estimation of the expected number of cases in each country was carried out adhering to a method proposed by Dorigatti et al. [13]. Let *C*_W_ be the cumulative number of plague cases in time window *W* in Madagascar. Expected number of Madagascar residents infected by *Yersinia pestis* and travelling to country *D* before the end of incubation or infectious periods in time window *W* was computed as

$E_{M,D}^{W}=C_{W}\frac{N_{D}}{365}\frac{T_{E}+T_{I}}{P_{M}}$, (8)

where *N*_D_ is the annual number of travellers to country D from Madagascar. *T*_E_ and *T*_I_ are the incubation and infectious periods of pneumonic plague. The incubation period was assumed to follow a lognormal distribution with mean 4.3 days and standard deviation (SD) of 1.8 days [14]. The infectious period was also assumed to follow a lognormal distribution with mean 2.5 days and SD 1.2 days [14]. *P*_M_ is the population size of Madagascar.

Similarly, the expected number of international travellers with pneumonic plague to home country O before the end of incubation and infectious periods is

$I_{O,M}^{W}=T_{O}\sum_{m=1}^{12}f_{m}p_{m}\frac{C_{W}}{P_{M}}\frac{T_{E}+T_{I}}{W}$, (9)

where *T*_O_ is the annual number of international travellers to Madagascar from country O. *f*_m_ and *p*_m_ are the proportion of international travellers visiting Madagascar in month *m* and relative proportion of the epidemic window *W* occurring in month *m*. Variability in the incubation and infectious periods was captured by randomly drawing their values from respective distributions for 10,000 times.

## Results for the estimated parameters

**Figure 2:**
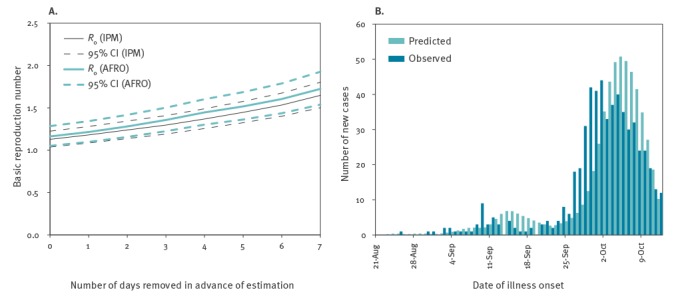
Transmission potential of primary pneumonic plague during the epidemic in Madagascar, August – October 2017

AFRO: World Health Organization Regional Office for Africa; CI: confidence interval; IPM: Institut Pasteur de Madagascar (IPM); *R*_0_: basic reproduction number

Using the observed epidemic curves from IPM and AFRO that were reported 31 October and 3 November, 2017, respectively, and assuming a constant reproduction number by 17 October 2017, *R*_0_ was estimated as shown in Figure 2A. Varying the length of days to remove the data from analysis for 0 to 7 days, the maximum likelihood estimate of *R*_0_ estimated based on the IPM dataset ranged from 1.12 to 1.65. Similarly, *R*_0_ estimated based on the AFRO dataset ranged from 1.16 to 1.72. Figure 2B compares the observed (IPM data) and predicted latest epidemic curves on 21 October 2017 employing an alternative model that explicitly addressed the reporting delay. *R*_0_ was estimated at 1.73 (95% confidence interval (CI): 1.55–1.95). Estimated mean length of reporting delay and variance were 6.52 days (95% CI: 5.55–7.57) and 20.69 day^2^ (95% CI: 13.21–31.68), respectively. The CFR was estimated at 5.49% (95% CI: 4.67–6.40).

(A) The basic reproduction number of pneumonic plague outbreak in Madagascar as estimated either from the epidemic curve reported on 21 October by IPM or the epidemic curve reported on 24 October 2017 by AFRO. Horizontal axis measures the number of recent days that were removed from our analysis due to reporting delay. Black straight and dashed lines represent *R*_0_ and its 95% confidence intervals estimated by IPM data and turquoise straight and dashed lines represent *R*_0_ and its 95% confidence intervals estimated by AFRO data. (B) Comparison between predicted and observed epidemic curves on 21 October 2017 reported by IPM. Time delay in reporting was captured by analysing multiple curves. Day 0 was set to be 23 August 2017 on which the index case developed fever.

**Figure 3:**
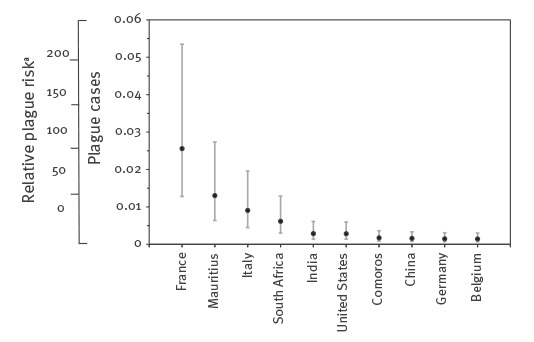
Expected number of pneumonic plague cases travelling from Madagascar, 1 August–31 October 2017

Figure 3 shows the expected number of cases travelling from Madagascar (i.e. summation of importation and exportation) from 1 August to 31 October 2017 (for a total of 92 days) with 95% tolerance interval (i.e. percentile estimates from 2.5^th^ to 97.5^th^ percentiles of random simulations), with the expected numbers for top-10 high risk countries. The highest number is less than 0.1 person, indicating that there is no strong indication of the high risk of international spread. The expected number in all other countries is given in the Online Appendix file [15]. While African countries in close proximity to Madagascar (e.g. Mauritius, South Africa and Comoros) showed relatively higher values, some populous countries (e.g. China, India and United States) and countries with high volume of travellers to Madagascar (e.g. France, Italy and Germany) were also estimated to be at risk.

Mean and 95% tolerance interval of the estimated number of cases travelling from Madagascar and the estimated relative risks (RR) during the outbreak from 1 August to 31 October 2017 (for a total of 92 days). Only the result of top-10 high risk countries is shown.

^a^ To calculate the RR, expected number of cases in France, the country at highest risk, was set as 100, so that the RR in other countries can be compared. Tolerance intervals were calculated based on 10,000 iteration samplings.

## Discussion

The present study is the first to comprehensively assess the early epidemiological dynamics of primary pneumonic plague epidemic in Madagascar in 2017 using several different mathematical models. *R*_0_ was estimated to range from 1.12 to 1.72 using single epidemic curves by IPM and AFRO reported on 21 October and 24 October 2017, respectively, and assuming a constant growth rate of cases. Using the IPM data, and explicitly accounting for the reporting delay which required the mean of 6.52 days, *R*_0_ was estimated at 1.73. CFR has been estimated to be as low as 5.5%. The expected number of imported cases in each country was estimated to be far less than the value of 1 person.

The transmissibility, *R*_0_, appeared to be consistent with, or slightly higher, than published estimates that rest on contact tracing data, including an epidemic in Madagascar in 2015 (*R*_0_ ranging from 1.1 to 1.3 and 1.4 in [14,16]), and a stochastic model-based estimate relying on cumulative incidence data from 1906 to 2006 (*R*_0_ ranging from 1.0 to 1.4 [17]). In our study, two different methods echoed each other to interpret the transmissibility. The transmissibility of the ongoing epidemic is not considerably different from earlier ones, and our analysis endorses that the transmissibility of primary pneumonic plague is in general not very high. Instead, due to the widespread number of human-to-human transmissible cases, the outbreak can reach geographic areas (cities) in Madagascar where there were no plague cases in earlier days. The CFR appeared to be smaller than a published estimate, e.g. reaching up to 70% [16]. By properly tracing contacts and bringing diagnosed individuals under appropriate antibiotic treatment, the estimate indicates that early treatment could prevent patients from fatal outcome.

As an additional finding, the risk of international spread was shown to be low. The expected number of imported cases of less than 0.1 person is much smaller than those calculated for Zika virus [13]. This can be mainly attributed to two facts. First, the absolute number of diagnosed plague cases has still remained below 3,000 cases, while Zika virus induced a far greater number of infections. Second, Zika virus infection involves asymptomatic presentation and also clinically mild cases, but plague is far more virulent than Zika virus requiring close medical attendance and treatment. There have been no confirmed cases outside Madagascar, and our study objectively endorsed that the risk of international spread is very low. Nevertheless, travellers should be made aware of the ongoing plague epidemic [10].

Two limitations must be noted. First, our modelling approach had to ignore the detailed heterogeneous transmission dynamics. For instance, spatial heterogeneity or more microgeographic insights have not been incorporated into our approach mainly due to limited information for implementing geospatial modelling. Moreover, contact heterogeneity, e.g. the role of superspreading events, has also not been fully captured. Second, we discarded datasets other than pneumonic plague. Although it is likely to be very rare, a bubonic plague case could develop to become a case of secondary pneumonic plague and cause a secondary transmission event to generate primary pneumonic plague cases. This issue can be addressed once clinical forms of all cases are clarified.

In conclusion, the transmission potential of pneumonic plague in Madagascar 2017 is not different from those in earlier pneumonic plague epidemics. The low CFR is potentially indicative of successful treatment outcome, and the risk of international spread is very limited.
